# Feet, heat and scallops: what is the cost of anthropogenic disturbance in bivalve aquaculture?

**DOI:** 10.1098/rsos.150679

**Published:** 2016-03-09

**Authors:** Anthony A. Robson, Lewis G. Halsey, Laurent Chauvaud

**Affiliations:** 1 LabexMER, UMS 3113 CNRS, Institut Universitaire Européen de la Mer, Université de Brest, Rue Dumont D’Urville, 29280 Plouzané, France; 2 Atmosphere and Ocean Research Institute, The University of Tokyo, 5-1-5 Kashiwanoha, Kashiwa, Chiba 277-8564, Japan; 3 Centre for Research in Ecology, Department of Life Sciences, University of Roehampton, Holybourne Avenue, London SW15 4JD, UK; 4 Laboratoire des Sciences de L’Environnement Marin (UMR CNRS 6539), Institut Universitaire Européen de la Mer, Technopôle Brest Iroise, 29280 Plouzané, France

**Keywords:** growth, temperature, anthropogenic disturbance, activity, metabolic rate, energy expenditure

## Abstract

The effects of unnatural disturbances on the behaviour and energetics of animals are an important issue for conservation and commercial animal production. Biologging enables estimation of the energy costs of these disturbances, but not specifically the effect these costs have on growth; a key outcome measure for animal farming enterprises. We looked at how natural and anthropogenically induced activity and energy expenditure of king scallops *Pecten maximus* varies with temperature. These data were then used to model growth time of king scallops reared in an aquaculture facility under different temperatures and anthropogenic disturbance levels. The scallops exhibited a typical total metabolic rate (MR)–temperature curve, with a peak reached at a middling temperature. The percentage of their total MR associated with spinning and swimming, behavioural responses to disturbance, was considerable. Interestingly, as temperature increased, the activity MR associated with a given level of activity decreased; a hitherto unreported relationship in any species. The model results suggest there is a trade-off in the ambient temperature that should be set by hatcheries between the optimal for scallop growth if completely undisturbed versus mitigating against the energy costs elicited by anthropogenic disturbance. Furthermore, the model indicates that this trade-off is affected by scallop size. Aquaculture facilities typically have controls to limit the impact of human activities, yet the present data indicate that hatcheries may be advised to consider whether more controls could further decrease extraneous scallop behaviours, resulting in enhanced scallop yields and improved financial margins.

## 1. Introduction

Anthropogenic disturbance of fished and farmed animals often manifests as unnatural stimuli such as noise, vibrations, chemical pollution, illumination and the casting of shadows. The cost of anthropogenic disturbances to farmed colonies, e.g. decreased somatic growth rates, decreased gamete production and fatalities [1,2] is likely to impact the financial viability of farming ectotherms, including reptiles, amphibians, molluscs and most fishes [3,4]. Mitigating such anthropogenic disturbances may minimize energetically costly non-feeding behaviours and hence maximize growth rates by reducing the energetic costs of such activities and increasing the time available to feed [3,4]. However, relatively little is known about the effect of anthropogenic disturbance on the behavioural time budgets and associated metabolic rate (MR) of most fished and farmed species [2,5,6]. Furthermore, for ectothermic species, ambient temperature is known to affect behaviour and MR, raising the question of whether and how temperature modulates the level of unnatural or anti-predator behaviour exhibited as a result of anthropogenic stimuli [7–9] and, ultimately, its effect on growth.

King scallops *Pecten maximus* inhabit waters along the Eastern Atlantic coast, from northern Norway south to the Iberian Peninsula, and are also distributed off the coast of West Africa and around the Azores, Madeira and the Canary Islands. King scallop meat is considered a luxury food and commands a high retail price. The species is farmed in Spain, France, Ireland, UK and Norway [10] with active hatchery–nursery production in several of those countries. Scallop growth in the wild is mainly regulated by water temperature rather than by the amount of food available [11,12]. In indoor aquaculture, their growth rates increase with temperatures above 10°C and continue to increase up to 23°C when food is provided ad libitum [13]. However, above 17–18°C, the condition index (the ratio of dry meat weight to dry shell weight [14]) is lower. The scallops exhibit a stress response at these high temperatures [13,15,16] probably, because these temperatures result in lower partial pressures of oxygen in their haemolymph [17], inducing rapid accumulation of calcareous shell deposits accompanied by poor tissue growth.

The accelerometry technique, which measures body motion to estimate behaviours and associated MR, has been established as a veritable method for obtaining the fine-scale behavioural time and energy budgets of a range of animals, including invertebrates [4,18–22]. In this study, we use the accelerometry technique to determine how temperature modulates the effects of anthropogenic stimuli on the behaviour and MR of king scallops in indoor aquaculture. We then use these data to model how much daily energy expenditure would be reduced if the scallops experienced no unnatural disturbances. Specifically, we model growth time against ambient temperature to two key shell heights under scenarios of different disturbance levels. We consider the optimal temperature for scallop growth if there is no human interference at all and also discuss how hatcheries could tailor their water temperature under the more realistic scenarios of various degrees of anthropogenic disturbance.

## 2. Material and methods

During 2012 and 2013, king scallops were collected by scuba divers from l’anse de Sainte-Anne (48.3579° N, −4.5488° W), Plouzané, France and placed in a sediment-lined tank filled with natural, unfiltered seawater at the Institut Universitaire Européen de la Mer (IUEM), France. In these and all other tanks and respirometry chambers used to maintain the scallops during the period of experimentation, the sediment provided was sterilized sand to a depth of 8 cm from l’anse du Dellec (48.3540° N, −4.5659° W), Plouzané. Data were collected only from scallops with ripe gonads, i.e. gonad maturity stages more than 5 to 6 determined using Mason’s gonad observation index [23] and that did not spawn before, between or during experiments. Seventy scallops were used in respirometry experiments to calibrate accelerometry data with rate of energy expenditure, and 66 scallops were placed in an aquaculture facility where their activity-time budgets and energy expenditure budgets were estimated from accelerometry data. The few scallops used in multiple conditions (see §§2.1 and 2.2) did not change their reproductive physiology (gonad maturity stage) or size (wet mass in air, volume and shell dimensions) during the period of experiments. This was because of the low temperatures these particular scallops were exposed to.

### 2.1 Respirometry-acceleration experiments

MR was measured in scallops as rate of oxygen (O_2_) consumption ($\dot{V}O_{2}$). MR was described as routine MR (RMR), activity MR (AMR) and total MR at each experimental temperature. RMR is defined as the MR of a quiescent, undisturbed scallop, which includes feeding behaviour and associated costs such as specific dynamic action, but specifically no movement [3,24–26]. AMR is the rate of energy expended specifically to undertake activity, and thus does not include the RMR component of MR during active periods. Because scallop metabolism includes a substantial anaerobic component, the repayment of oxygen debt after a period of activity had finished was also measured. Total MR was calculated as mean MR across the entire recording period, and AMR was calculated as total MR minus RMR [24,27]. Activity level was measured from accelerometry as amount of dynamic body movement, calculated as the vector sum of dynamic body acceleration recorded in the three dimensions: VeDBA (*g*) [18,28–30]. $\dot{V}O_{2}$

and VeDBA were recorded for three filter-feeding scallops simultaneously using instrumented data loggers and a custom-made three-channel O_2_ respirometry set-up. On the morning that the experiment was to begin, three animals were placed individually into one of three Perspex respirometry chambers. The chambers were cylindrical, transparent, flat-bottomed and with a domed lid (maximum internal diameter: 19.5 cm, maximum internal height: 22.0 cm), and contained sterilized sand. The respirometry system contained 4.67 l of seawater when an animal was not present. Water flow rate in each respirometry chamber was 2 l min^−1^, allowing stable measurement of O_2_ concentration by a calibrated dissolved O_2_ probe (Seaguard^r^O_2_ optode 4835, AADI, Bergen, Norway) sampling once every 2 s. O_2_ concentration was measured by intermittent flow-through respirometry using an automatic water changer (custom-made by Stephen P. Uphill, Stockport, UK) to periodically re-oxygenate the water and then reseal the system to allow continuation of the O_2_ measurement. The O_2_ level within each respirometer chamber was not allowed to drop below 75% of saturation [31]. The lag time of the system was determined by bleeding CO_2_ into the water [22]. Background $\dot{V}O_{2}$ in the water owing to aerobic organisms in the sediment was measured and accounted for in calculations of scallop MR along with the decrease in background $\dot{V}O_{2}$ over time owing to scallop filter-feeding which was determined by linear interpolation.

Scallop activity was recorded using acceleration data loggers (AXY-2, TechnoSmArt, Italy) set to record acceleration in three axes (0–4 *g*), and temperature (°C), at 25 Hz onto a 1 Gb internal memory chip. This recording frequency was sufficiently high to use measures of organism-induced acceleration as a proxy for MR and to clearly ascertain when an animal was active [3,32]. Preset loggers were wrapped in cling film and waterproofed using adhesive-lined heat shrink tubing. Epibionts were removed from the outer shell surface of all subject animals. Industrial strength Velcro was glued to the outer shell of the upper (left/flat) valve of each scallop and to each data logger using cyanoacrylate, as the means for attaching the logger to the scallop.

Including glue and Velcro, the loggers instrumented to scallops had a mass in air of 2.99 g and a volume of 3.5 ml. The air trapped inside the waterproofed loggers made them neutrally buoyant in seawater. Data from the acceleration data loggers were downloaded onto a PC using AXY Manager software (TechnoSmArt, Italy). First viewing these data in OriginPro (v. 9.1, OriginLab Corporation, USA), periods of scallop activity were defined as those with a minimum duration of 0.16 s (four recorded data points) that had a mean VeDBA of at least 0.05. Then, from these data, periods of activity and the associated values of VeDBA for scallops were accurately determined using the custom-made R [33] package, BEnergetix [24,34]. Scallops typically move for less than 2 min per day [3], so the slight inherent background noise in the accelerometry measures can result in overestimates of mean VeDBA over all periods of activity across the duration of the recording. To rectify this, during periods of inactivity, VeDBA values were set to 0, and thus VeDBA, when scallops were not moving, is reported as 0.

To determine the effect of temperature on scallop activity and MR, the respirometry chambers were placed within an opaque grey sediment-lined tank (3×2×0.9 m deep) filled with seawater (salinity 32–33‰), in a temperature-controlled room on a 12 : 12 h light : dark cycle. The lights turned on at 07.00 and off at 19.00. The seawater in the scallop tank was continuously exchanged at a rate of 750 l h^−1^ with fresh unfiltered seawater from temperature-controlled reservoir tanks.

Scallops in sediment-lined tanks were exposed to seven different experimental temperatures, in a preset randomized order: 15, 9, 6, 12, 18, 24 and 21°C. Scallops were exposed to changes in experimental temperature at rates of no greater than 1°C per day and then maintained at the experimental temperature for at least four weeks before data were recorded such that they were fully acclimatized [15,35]. Before, during and between experiments, scallops were provided a natural diet (unfiltered seawater) including seston, dissolved matter and benthic particulate matter. Scallops were exposed to chlorophyll *a* concentrations (a proxy for scallop food concentration) similar to those present in the Rade de Brest. At 6–9 and 12–24°C, mean ± the 95% confidence interval (CI) chlorophyll *a* concentration was 0.62±0.16 and 1.40±0.15 μg l^−1^, respectively. Data were collected from 10 to 13 scallops at each temperature, with a few individuals used in multiple temperature conditions.

The scallops were instrumented with the acceleration data logger and then placed in individual respirometry chambers 11 h prior to the start of data collection. In each temperature condition, scallop data were recorded for 10 h overnight, in the dark. $\dot{V}O_{2}$ was calculated using the following equation:

$$\dot{V}O_{2}=([O_{2}]t_{0}-([O_{2}]t_{1})\times \left(\frac{V-aV}{t}\right)-\left(\frac{\mathrm{background}\;\dot{V}O_{2}}{V}\times (V-aV)\right),$$

where $\dot{V}O_{2}$ is the rate of O_2_ consumption (mg $O_{2}\min^{-1}$), [O_2_]*t*_0_ is the O_2_ concentration at time *t*_0_ (mg O_2_ l^−1^), [O_2_]*t*_1_ is the O_2_ concentration at time t_1_ (mg O_2_ l^−1^), *V* is the respirometry system volume (l), *aV* is the volume of the logger-instrumented scallop (l), *t*=*t*_1_−*t*_0_ (min) and background $\dot{V}O_{2}$ is the rate of background O_2_ consumption (mg $O_{2}\min^{-1}$).

Scallop RMR, AMR and total MR were calculated from the recordings of $\dot{V}O_{2}$ using R and MS Excel. At each temperature, RMR was the lowest mean MR value calculated over 20 consecutive minutes within a 10 h dataset, which was always when there were no period(s) of raised $\dot{V}O_{2}$ associated with scallop movement. Because the groups of scallops in each temperature condition were mostly comprised different individuals, scallop data are presented as ash-free dry tissue mass-corrected values, using a mass scaling exponent of 0.52 calculated from a mixed linear model of scallop MR against ash-free dry tissue mass, in which temperature did not interact with ash-free dry tissue mass.

### 2.2 Twenty-four hour aquaculture facility experiments

A different set of scallops to those used in the respirometry experiments were also placed within sediment-lined tanks. All other environmental details are also the same as described above.

Temperature-acclimatized scallops were exposed to nine different experimental temperatures, in a preset randomized order: 13, 10, 6, 15, 9, 11, 18, 24 and 21°C. At 6–9 and 10–24°C, mean ± 95% CI chlorophyll *a* concentration was 0.69±0.16 and 1.31±0.13 μg l^−1^, respectively. Data were collected from 10 to 12 scallops at each temperature, with a few individuals used in multiple temperature conditions.

The scallops were instrumented with an acceleration data logger and then allowed to settle in the tank for one week, inside a locked aquaculture facility without human presence [36], before data were collected. At each temperature, scallop data were recorded for 24 h. From the data collected in the respirometry-acceleration experiments, the RMR of the scallops in the aquaculture facility was estimated from ash-free dry tissue mass using the equation

2.1 log10 RMR (mg O2min−1)=(0.523×log10 ash-free dry tissue mass (g))+(0.030×temperature (C∘))−2.504r2=0.89, p<0.001.

Given that scallop activities are mainly anaerobic, $\dot{V}O_{2}$ can only be used to estimate rate of energy expenditure during activity by measuring $\dot{V}O_{2}$ raised above RMR both immediately before, during and immediately after the activity until full recovery (MR=RMR). For full details of how AMR was calculated from the respirometry data, see ([3] and the electronic supplementary material, S2 in [24]). Most importantly, $\dot{V}O_{2}$ above RMR immediately after the cessation of activity until it decreased to within +1 standard deviation of RMR was included in the calculations of AMR; this period of raised $\dot{V}O_{2}$ typically lasted between 0.5 and 30 min.

AMR was estimated from VeDBA using the equation

2.2 log10 AMR (mg O2 min−1)=(0.702×log10 VeDBA (g))+0.976×log10 ash-free dry tissue mass (g))−(0.013×temperature (C∘))+1.257r2=0.85, p<0.001.Estimated total MR=estimated RMR+estimated AMR.

Estimated scallop MR data are presented as ash-free dry tissue mass-corrected values, using a mass scaling exponent of 0.52. Infrared video camera recordings of sampled scallop behaviour confirmed that BEnergetix accurately classified each scallop movement as either a cough, dig, turn, 180° flip, spinning event or swim as well as accurately determining the duration of each behaviour and thus its associated mean VeDBA. Unless stated otherwise, means are reported ± the 95% CI. Sometimes, visualization of these statistics is used to infer evidence of differences between temperature conditions [37–40]. Variables were log_10_ transformed prior to regression analysis where appropriate.

### 2.3 Modelling growth rate against temperature in scenarios of different disturbance levels

Where energy is expended by scallops on activities in response to disturbance, energy is deemed lost that would otherwise be used for growth. This premise forms the basis of the models on growth rate described as follows.

We combined data on energy expenditure recorded in this study with data on king scallop growth rates at different sizes and ambient temperatures presented in [13,15] to estimate changes in rates of scallop growth owing to human-induced behaviours. These growth rate studies report some anthropogenic disturbance in the aquaculture facility where the experiments took place [15,41] and thus, we assume disturbance levels during those studies were the same as for our indoor aquaculture experiments.

We modelled: (i) the effect on scallop growth if there was no anthropogenic disturbance (i.e. spinning and swimming AMR=0), (ii) the effect on scallop growth at the level of anthropogenic disturbance observed in this study, and (iii) the effect of even greater anthropogenic disturbance (defined as extra swims per day) on scallop growth than observed in this study [13,15]. Growth rates were calculated on a per day basis.

Additional AMR owing to extra swims was estimated using the AMR equation (2.2) with a swim mean VeDBA of 0.66 *g* and mean single swim duration of 5.2 s. ANOVA provided no evidence for an effect of temperature (10–18°C) or scallop ash-free dry tissue mass on swim VeDBA or single swim duration: all *p*≥0.60. The two scenarios were both modelled over two size ranges: for shell height growth from 10 to 60 mm, because at 60 mm scallops are less susceptible to predators such as crabs and starfish and thus start to be cultivated on the seabed [13]; and for shell height growth from 10 to 130 mm, because 130 mm represents the size of a large hatchery scallop used as broodstock. Data on the daily increase in shell height in relation to scallop size and ambient temperature are provided by Laing [13,15].

Shell height was converted to ash-free dry tissue mass using the following equation derived from data in this study:

2.3 $$\begin{array}{rl} \log_{10}\;\text{ash-free dry tissue mass}\;(g) & =(3.487\times \log_{10}\;\text{shell height}\;\left(\mathrm{mm}\right))\\ & \;-5.995\;(r^{2}=0.98,\;p<0.001). \end{array}$$

One gram of total dry tissue mass of scallop (24.5 kJ of energy; [42]) was converted to 1 g ash-free dry tissue mass (29.8 kJ) using the following relationship between scallop total dry tissue mass and ash-free dry tissue mass derived from data in this study:

2.4 $$\begin{array}{rl} \text{total dry tissue mass (g)} & =(1.197\times \text{ash-free dry tissue mass}\;\left(g\right))\\ & \;+0.151\;(r^{2}=0.97,p<0.001). \end{array}$$

Using a mean absorption efficiency of 0.47 [31], 1 g of ash-free dry tissue mass growth requires 63.4 kJ of food ingestion. In turn, it was assumed that each 63.4 kJ of energy expended owing to AMR (mg O_2_ d^−1^; estimated using the AMR equation (2.2)) associated with swimming or spinning as a result of anthropogenic disturbance represented a reduction in growth of 1 g ash-free dry tissue mass. AMR measured as mg O_2_ min^−1^ was converted to kJ using the conversion factor of 19.9 J ml^−1^ O_2_ [43], taking account of seawater temperature and depth.

Daily growth rate was calculated by dividing the estimated gain in ash-free dry tissue mass associated with shell height growth from 10 up to 60 or 130 mm by the time estimated for the shell to reach that height [13,15]. The mean ash-free dry tissue mass within each shell height range investigated by Laing [13,15] was included in equation (2.2) alongside temperature and VeDBA for estimating the ‘lost ash-free dry tissue mass’ per day owing to anthropogenic disturbance.

To model scenario (i) the daily loss in ash-free dry tissue mass was added to the predicted growth rate, and to model scenario (iii) the daily ash-free dry tissue mass loss was subtracted from the predicted growth rate. To model scenario (ii), no adjustments to predicted growth rate were required. The predicted number of days to reach 60 or 130 mm shell height in each scenario was then calculated.

## 3. Results

The mean wet mass of a scallop in air and its volume in water with epibionts (*n*=136) was greater than for the same scallops with the epibionts removed and instrumented with a logger (mean mass: 179.5±13.9 and 173.0±12.9 g, respectively; mean volume: 116.6±9.4 and 112.7±8.7 ml, respectively). Scallop ash-free dry tissue mass, shell height (maximum distance from umbo to shell edge), maximum shell length (parallel to the hinge) and volume ranges were: 1.97–18.83 g, 65–125 mm, 73–143 mm and 29–213 ml, respectively. In the Rade de Brest, from where the scallops were collected, the annual mean ± s.d. seawater temperature (1998–2012 weekly mean data pooled) was 13.37±2.88°C.

### 3.1 Scallop behavioural energetics and temperature

**Figure 1.**
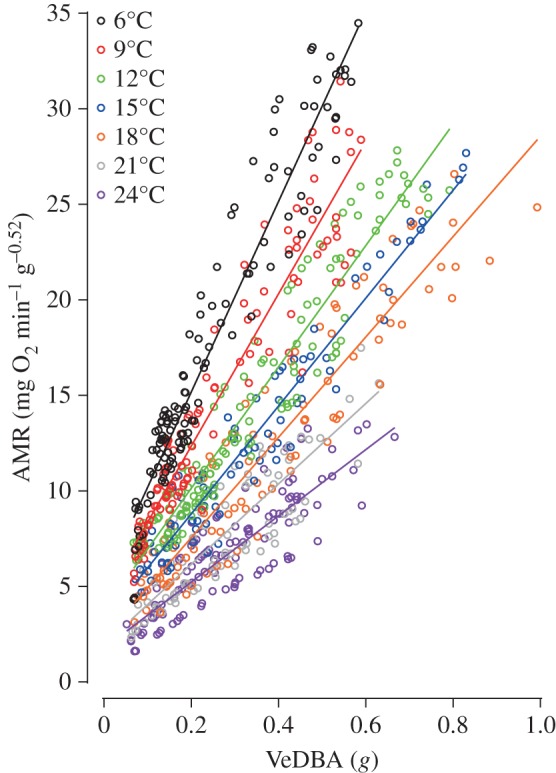
Activity metabolic rate (AMR) against VeDBA (*g*) in king scallops at different temperatures denoted by different colours. Best-fit regression line *r*^2^ range: 0.72–0.92.

**Figure 2.**
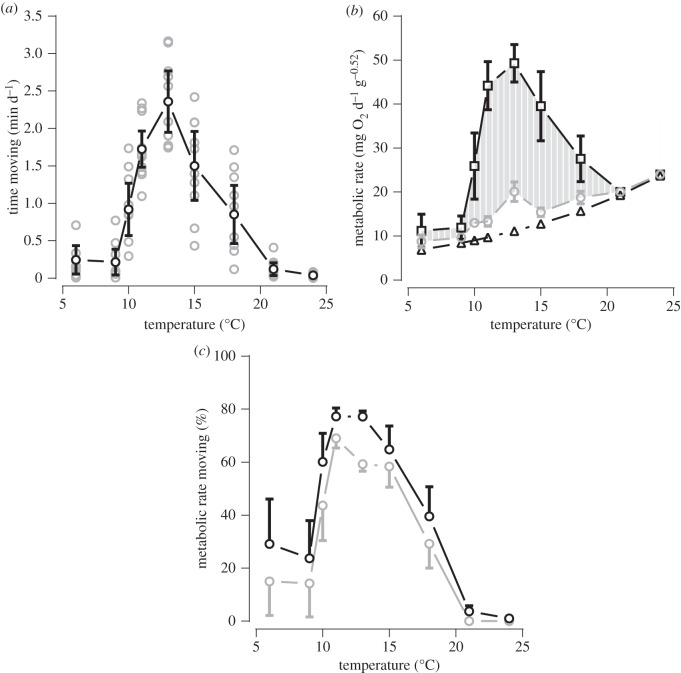
King scallop (*n*=10–12 at each temperature) activity levels and estimates of metabolic rate against temperature. (*a*) Time scallops spent moving per day (each data point (grey circle) at each temperature represents a measured value from one individual); (*b*) routine metabolic rate (RMR; triangles), total metabolic rate (total MR; squares) and total MR excluding that associated with spinning and swimming (circles) per day—the grey-shaded area represents the magnitude of spinning and swimming activity metabolic rate (AMR); and (*c*) the percentage of total MR attributed to AMR (black) and to only spinning and swimming components of AMR (grey) against temperature. In each panel, means at each temperature are presented + and/or −95% confidence intervals (adapted for clarity).

Visual inspection of the data suggested no influence of rhythms on the movement and associated MR of the scallops. Plots of measured AMR against activity level (VeDBA; *g*) during a range of king scallop movements clearly showed that as temperature increases the energetic cost of any given level of activity decreases (figure 1). A full factorial general linear model including VeDBA and temperature with scallop identification as a random factor confirmed this, providing strong evidence that the interaction term was an important predictor of AMR (*F*_1,681_=484.96, *p*<0.001). In the aquaculture facility experiments, there was a clear peak at 13°C in the mean amount of time scallops spent moving (2.36 min d^−1^), with negligible but never zero movement at 6, 9, 21 and 24°C (figure 2*a*). Mean estimated total energy expended per day also peaked at 13°C (49.3 mg O_2_ g^−0.52^; figure 2*b*). Mean estimated total energy expended at 13°C excluding the estimated cost of spinning and swimming (the behaviours owing to anthropogenic disturbance) was 20.1 mg O_2_ g^−0.52^ d^−1^, which was greater than at all other temperatures except at 24°C (24.0 mg O_2_ g^−0.52^ d^−1^; figure 2*b*). At most temperatures (6–18°C), anthropogenic disturbance caused king scallops to spend more time (range: 51.9–85.4%) and energy (56.4–91.4%) on spinning and swimming than on coughing, digging, turning and flipping (figure 2*c*). Indeed, removing the cost of spinning and swimming behaviours (represented by the grey-shaded area in figure 2*b*) from estimated total energy expenditure dramatically reduced the mean total energy expended from between about 9 and 31 mg O_2_ g^−0.52^ d^−1^ depending upon the temperature (10–18°C; figure 2*b*). The percentage of estimated total MR attributed to spinning and swimming was on average 44% at 10°C, higher than 50% at 11, 13 and 15°C, 29% at 18°C and zero at 21 and 24°C (figure 2*c*). The overall message from these data is that across a wide range of temperatures, behaviours caused by anthropogenic disturbance can constitute a considerable amount of the total energy expended per day by a farm-reared king scallop. However, at high and low temperatures, where movement levels are low, anthropogenic disturbance has a heavily attenuated effect on total MR.

### 3.2 Modelling growth rate

**Figure 3.**
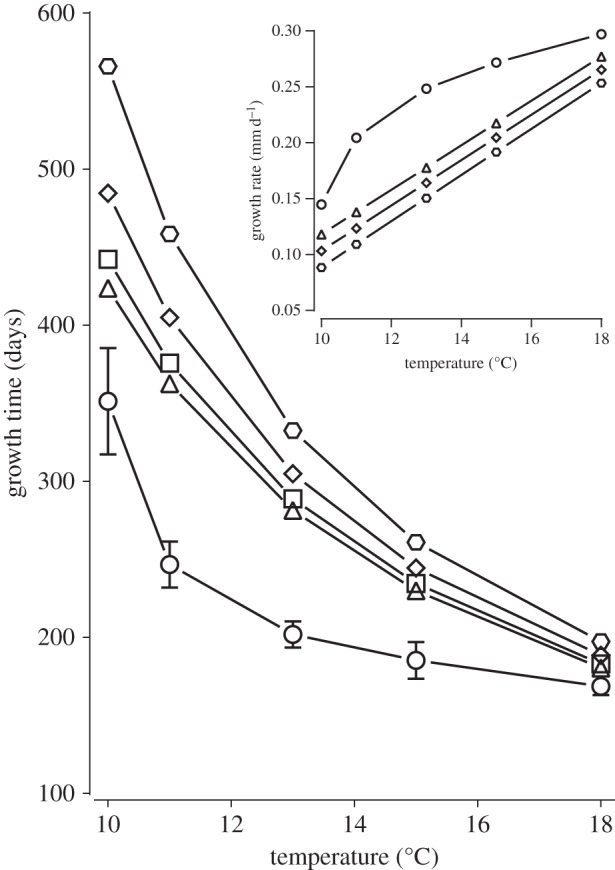
Modelling the effect of anthropogenic disturbance on the mean time for king scallops to grow from 10 to 60 mm in shell height, including the modulation of this effect by seawater temperature. Triangles: measured values for scallops exposed to the levels of anthropogenic disturbance observed in the present study; data taken from [13,15]. Circles: modelled values for scallops theoretically exposed to zero anthropogenic disturbance; the accompanying error bars indicate ±95% CI of the estimate. Other symbols: modelled values for scallops exposed to greater anthropogenic disturbance than observed in the present study [13,15]: theoretically exhibiting one (squares), three (diamonds) and six (hexagons) extra swims per day. Inset: mean rate of shell growth against temperature from 10 to 60 mm in shell height. Triangles: scallops exposed to levels of anthropogenic disturbance observed in this study; data taken from [13,15]. Circles: modelled scallops exposed to zero anthropogenic disturbance. Diamonds and hexagons: scallops theoretically exposed to greater anthropogenic disturbance than observed in this study [13,15] resulting in three and six extra swims per day, respectively.

Growth rate at the lowest (6 and 9°C) and highest (21 and 24°C) temperatures recorded in this study are not modelled, because the population of scallops studied do not grow at very low temperatures and they exhibit a stress response at high temperatures [13,15]. The model indicates that regardless of the degree of anthropogenic disturbance, the mean time for hatchery scallops to grow from 10 to 60 mm shell height (at 60 mm scallops start to be cultivated on the seabed) exhibits a negative, monotonic relationship with temperature (figure 3 main). However, there is an interaction between the degree of anthropogenic disturbance and temperature. At higher temperatures, different levels of anthropogenic disturbance have little effect on growth time to 60 mm shell height (e.g. mean growth time is 183, 188 and 197 days with one, three and six extra swims per day at 18°C, respectively), whereas at lower temperatures, the effect of the degree of anthropogenic disturbance is considerable (e.g. 422, 485 and 566 days at 10°C). Further, as temperature increases, the estimated reduction in growth rate caused by extra swimming in response to greater anthropogenic disturbance attenuates. This is shown as a gradual convergence of the lines of relationship between growth rate and temperature in scenarios of ever-greater anthropogenic disturbance (figure 3 inset). At 10°C, the decrease in growth rate when scallops are exhibiting three and six extra swims per day owing to extra anthropogenic disturbance is 0.015 and 0.030 mm d^−1^, respectively (a 13% and 25% decrease), whereas at 18°C, the decrease in growth rate is 0.012 and 0.024 mm d^−1^, respectively (a 4% and 9% decrease).

**Figure 4.**
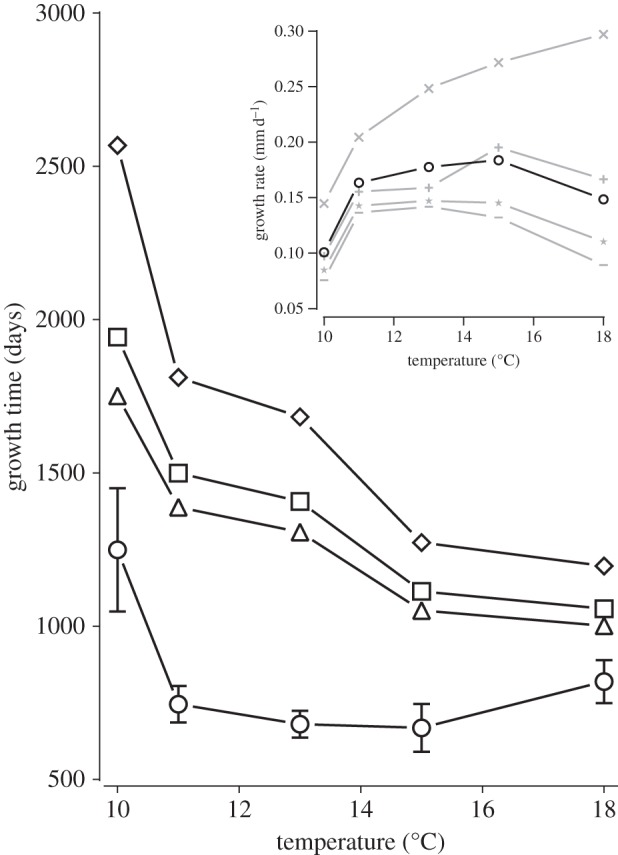
Modelling the effect of anthropogenic disturbance on the mean time for king scallops to grow from 10 to 130 mm in shell height, including the modulation of this effect by seawater temperature. Triangles: measured values for scallops exposed to the levels of anthropogenic disturbance observed in the present study; data taken from [13,15]. Circles: modelled values for scallops theoretically exposed to zero anthropogenic disturbance; the accompanying error bars indicate ±95% CI of the estimate. Other symbols: modelled values for scallops exposed to greater anthropogenic disturbance than observed in the present study [13,15]: theoretically exhibiting one (squares) and three (diamonds) extra swims per day. The inset presents the modelled mean growth rate of scallops exposed to zero anthropogenic disturbance against temperature from 10 to 130 mm in shell height (circles), and subsets of this growth range denoted by grey symbols: 10–60 (exes; data repeated in figure 3 inset), 60–80 (pluses), 80–100 (stars) and 100–130 (dashes).

The results are qualitatively similar when considering the growth of scallops up to 130 mm shell height (hatchery scallop broodstock size), although there are also some notable differences (figure 4). Figure 4 inset presents only the scenario of no anthropogenic disturbance, and includes data showing that as scallop size increases growth rate decreases. Figure 4 inset also shows that although mean growth rate up to 130 mm shell height increases as temperature increases up to 15°C, it decreases by 18°C. Thus, when anthropogenic disturbance is zero, scallops grow to 130 mm most quickly in 15°C seawater (figure 4 main). Similar to the model for growth to just 60 mm shell height, different levels of anthropogenic disturbance interact with temperature to affect growth time, with the degree of anthropogenic disturbance having a bigger effect at lower temperatures. However, in contrast to the previous model, at the highest temperatures, growth time still varied considerably with the level of anthropogenic disturbance (growth times ranging from 1057 to 1196 days with one and three extra swims per day at 18°C, respectively; figure 4 main).

## 4. Discussion

The effect of non-lethal unnatural disturbances on the behavioural energetics of animals is an important issue for conservation, fisheries, farming and wider ecosystem management [44]. Biologging has been used to estimate the energy costs of these disturbances [3], but not specifically the effect that these energy costs have on growth, which is a key outcome measure for animal farming enterprises. This study looks at how both natural and anthropogenically induced activity and energy expenditure of king scallops varies with temperature. These data are then applied to model growth time of scallops reared in an aquaculture facility to two industry-relevant sizes under scenarios of different temperatures and anthropogenic disturbance levels.

### 4.1 Effect of temperature on scallop behavioural energetics

King scallops exhibit a fairly typical total MR–temperature curve, with a peak reached at a middling temperature (in this case, around 13°C) and markedly lower total MR at relatively low and high temperatures ([27]; figure 2). The proportion of time spent moving approximately tracks total MR, particularly at lower temperatures, again peaking at around 13°C. Interestingly, as temperature increases, the AMR of the scallops to perform a given level of activity decreases (figure 1). To the best of our knowledge, this relationship between activity-specific energy efficiency and temperature has not been reported in any animal previously, perhaps because relationships presented between MR and temperature do not usually partition resting and active costs [27]. The relationship may be explained by the recent findings of Seebacher *et al*. [45], who reported that higher temperatures relate to lower oxygen consumption per unit work in muscle owing to decreased muscle viscosity and stiffness.

Thus, 13°C appears to be a fairly specific optimum temperature [46] for king scallops, at least for the population sampled in this study. Towards the highest and lowest temperatures to which the scallops were exposed, there was little (but never zero) movement (figure 2*a*). Low activity levels at the lowest temperatures are generally explained by the lack of heat present to ensure sufficient temperature-dependent biochemical reactions within the body in order to support higher levels of activity [47,48]. Less activity by the scallops at the highest temperatures is perhaps most likely owing to a reduced level of force generated by the muscles [17,49]. Given that once ambient temperature has reached 13°C total MR peaks (figure 2*b*), perhaps most plausible is that at this temperature the rate of maximum oxygen delivery by the scallops has been reached. In turn, as temperature rises further, RMR continues to increase therefore less oxygen is available for activity and recuperation from activity, exacerbated by the lower oxygen levels present in warmer water [50], resulting in a decrease in activity levels. Alternatively, scallops might move less at high temperatures because of a loss of nervous function that impairs activity [51].

Measured as time spent moving, scallops exhibited a transition between low activity and high activity at around both 10 and 18°C (figure 2*a*). The sensitivity of the scallops to water temperature may well be explained by the fact that subtidal species typically experience much slower and smaller variations in ambient temperature in their natural environment compared with intertidal species [52]. A similar pattern between total daily energy expenditure and temperature was shown for bay scallops *Argopecten irradians concentricus* albeit across just four temperatures [53]. The average percentage of total MR allotted to AMR was highest at 11 and 13°C (figure 2*c*). At those temperatures, there is high oxygen availability to support activity and recuperation from activity because of high oxygen delivery to the tissues and higher oxygen levels present in cooler seawater. Of course, all activities incur energetic costs, and for king scallops, their spinning and swimming behaviours, triggered by anthropogenic disturbance, are the most expensive [3]. The percentage of total MR allotted to AMR for spinning and swimming by the scallops in this study was considerable within the temperature range where these activities were exhibited (figure 2*c*), representing more than 50% of their energy costs spent on activity at temperatures between 11 and 15°C, inclusive.

### 4.2 Scallop growth models

Considering hatchery scallops during growth up to 60 mm shell height (at 60 mm scallops start to be cultivated on the seabed), they grow more quickly at higher temperatures, and this trend holds, regardless of the degree of anthropogenic disturbance they are exposed to. Thus, their growth time to 60 mm is shorter at higher temperatures (figure 3 main). RMR is higher at greater temperatures (figure 2*b*), which may enable more rapid tissue accumulation [13,15,16]. Furthermore, scallops tend to exhibit lower activity levels at higher temperatures (figure 2), and this combined with the modulating effect of temperature on the relationship between AMR and activity level (figure 1) means that anthropogenic disturbance results in less of an increase in energy expenditure in warmer water. Owing to these lower energy expenditures in response to anthropogenic disturbance at higher temperatures, in combination with the increased growth rates, the differences in growth times to 60 mm between scallops exposed to different levels of anthropogenic disturbance become less pronounced at higher temperatures (figure 3 main). The highest temperature modelled in this study was 18°C, which shows the greatest attenuation of anthropogenic disturbance on the time taken for a scallop to grow to 60 mm. However, at this temperature, the condition index of scallops may be impaired, because scallops exhibit a stress response resulting in poor tissue growth relative to shell growth [13,15,16]. This may explain why the recommendation based on observation is to rear king scallops at 17°C [15].

For any given temperature, the effect of increased anthropogenic disturbance on growth times was greater in the scallops when grown up to broodstock size (130 mm in shell height) compared with only 60 mm, although this effect was still attenuated at the highest modelled temperature of 18°C (figure 4 main). This is because, as would be expected, the absolute energy costs to exhibit a given behaviour are greater in larger individuals (see equation (2.2)) and thus anthropogenic disturbance has a greater toll on energy expenditure. In all cases where a degree of anthropogenic disturbance was present, similar to the model to 60 mm shell height, growth time to 130 mm was quicker at higher temperatures. However, in contrast to growth up to just 60 mm, growth rates of scallops to 130 mm exposed to zero anthropogenic disturbance do not follow a positive monotonic relationship with temperature (figure 4 inset). Instead, growth rate reaches a plateau between around 11 and 15°C. This more complex relationship arises because as the scallops grow larger, the difference in shell growth rate between temperatures decreases [13], and this attenuated effect of higher temperature on growth rate is overcome by the associated higher absolute AMR. Thus, while small scallops exhibit their highest growth rates at the highest temperatures, progressively larger scallops in the scenario of no anthropogenic disturbance exhibit an ever more accentuated downturn in growth rate beyond around 15°C. When anthropogenic disturbance is included in the model, at 18°C, AMR is higher than in the scenario of no anthropogenic disturbance but relatively low compared with the AMR when anthropogenic disturbance is modelled at temperatures up to around 8°C less than 18°C (figure 2). This large reduction in AMR owing to anthropogenic disturbance across this range of temperatures ensures that growth time consistently decreases as temperature increases (figure 4 main).

### 4.3 Implications for aquaculture

Simply approaching a tank of scallops causes the majority of animals to swim, even when they are in complete darkness (A. A. Robson, M. S. Kelly 2003–2004, personal observation via infrared camera); cultured scallops are very sensitive to anthropogenic disturbance. During the experiments in this study, in contrast to many indoor aquaculture facilities, scallops were not exposed to direct human contact [3]. However, similar to other aquaculture facilities, the one used in this study was not soundproof. For example, there were vibrations caused by the presence of people near the aquaculture facility and from the water pump inside the aquaculture facility, as well as the unnatural lighting and environment, all of which contributed to the experimental scallops experiencing anthropogenic disturbance.

Some commercial enterprises will set the temperature at that which they consider to be the growth rate optimal [13,15], or such that the overhead costs are minimized, whereas others accept the ambient temperature to avoid heating costs. A balance between the two strategies is sometimes used [13,54–57]. The results presented here suggest there is a trade-off in the ambient temperature that should be set by hatcheries between the optimal for king scallop growth in a disturbance-free scenario versus mitigating against the energy costs elicited by anthropogenic disturbance. The results further indicate that those optima differ with scallop size. For growing scallops to only 60 mm in shell height, growth time is minimized at higher temperatures, particularly when anthropogenic disturbance is greater, i.e. higher temperatures result in shorter growth times, and this is particularly the case (temperature has a particularly pronounced effect) when anthropogenic disturbance is high. For growing scallops to broodstock size, optimal temperature in terms of growth time may again be high, but where anthropogenic disturbance is low, slightly lower temperatures may be optimal, and of course have the added advantage of reducing heating costs. In the future, the accuracy of the growth models presented in this study could be tested by empirical validation, with experimental condition described by different degrees of anthropogenic disturbance.

Some aquaculture facilities already have fairly strict controls on human activities. Nonetheless, they may be advised to consider whether further decreases in extraneous scallop behaviours might be possible through further additional reductions in anthropogenic stimuli. In indoor aquaculture, exposing scallops to natural light, soundproofing the facilities, employing polyculture (a more natural environment including natural sounds [58]) and keeping human contact to a minimum could be steps towards reducing energetically costly non-feeding behaviours [3]. The negative effects of disturbance on growth are undoubtedly applicable to many other ectothermic species including animals that are exposed to unnatural noise/vibrations, chemical pollution, artificial illumination at night, boat wakes and wind gusts caused by passing vehicles.

## Supplementary Material

Excel file: Summary and detailed daily behavioural time budgets and associated metabolic rate of hatchery scallops from 6 to 24 degrees Celsius.

## Data accessibility

The latest beta version of BEnergetix is available from A.A.R. The datasets supporting this article have been uploaded as part of the electronic supplementary material.
